# Enhancement of the Si *p-n* diode NIR photoresponse by embedding *β*-FeSi_2_ nanocrystallites

**DOI:** 10.1038/srep14795

**Published:** 2015-10-05

**Authors:** A. V. Shevlyagin, D. L. Goroshko, E. A. Chusovitin, K. N. Galkin, N. G. Galkin, A. K. Gutakovskii

**Affiliations:** 1 Institute of Automation and Control Processes FEB RAS, Radio St. 5, 690041 Vladivostok, Russia; 2 Far Eastern Federal University, School of Natural Sciences, Sukhanova St. 8, 690950 Vladivostok, Russia; 3 Rzhanov Institute of Semiconductor Physics, SB RAS, Lavrentieva Ave.1, 630090 Novosibirsk, Russia; 4 Novosibirsk State University, Pirogova St. 2, 630090 Novosibirsk, Russia

## Abstract

By using solid phase epitaxy of thin Fe films and molecular beam epitaxy of Si, a *p*^+^-Si/*p*-Si/*β*-FeSi_2_ nanocrystallites/*n*-Si(111) diode structure was fabricated. Transmission electron microscopy data confirmed a well-defined multilayered structure with embedded nanocrystallites of two typical sizes: 3–4 and 15–20 nm, and almost coherent epitaxy of the nanocrystallites with the Si matrix. The diode at zero bias conditions exhibited a current responsivity of 1.7 mA/W, an external quantum efficiency of about 0.2%, and a specific detectivity of 1.2 × 10^9^ cm × Hz^1/2^/W at a wavelength of 1300 nm at room temperature. In the avalanche mode, the responsivity reached up to 20 mA/W (2% in terms of efficiency) with a value of avalanche gain equal to 5. The data obtained indicate that embedding of *β*-FeSi_2_ nanocrystallites into the depletion region of the Si *p-n* junction results in expansion of the spectral sensitivity up to 1600 nm and an increase of the photoresponse by more than two orders of magnitude in comparison with a conventional Si *p-n* junction. Thereby, fabricated structure combines advantage of the silicon photodiode functionality and simplicity with near infrared light detection capability of *β*-FeSi_2_.

Light detection in the near-infrared region (NIR), especially at the telecommunication wavelengths (1300 and 1550 nm), is an important issue for optical fibre communication systems because of the low dispersion and low loss characteristics of the silica fibres^1^. To step into the future of integrated optoelectronics, we need light sources and detectors that are compatible with conventional Si technology. That is why, despite the success of applications of, for example, A_III_B_V_-based photodetection materials, such as InAs/GaSb/AlSb superlattices^2^,^3^, their utilization is limited. On the other hand, in recent years there has been considerable progress in terms of the development of photodetecting structures on Si substrates. Among them are other group IV materials and their alloys, quantum dots, or wells, namely GeSn *p-i-n*^4^,^5^ or SiGe structures^6^,^7^,^8^, and graphene-based photodetectors grown on Si^9^,^10^. The common drawback of these structures is the absence of effective light sources based on the very same materials.

Therefore, the semiconducting iron disilicide (*β*-FeSi_2_) has the advantage of the ability to simultaneously emit and detect light. Semiconducting iron disilicide exhibits a band gap of approximately 0.8 eV and a very large optical absorption coefficient of over 10^5^ cm^−1^ at 1 eV^11^. It was shown that the Si/*β*-FeSi_2_ heterostructure diode possesses electroluminescence at a wavelength of 1550 nm at room temperature with an external quantum efficiency near to 0.1%^12^. Hence, the development of Si/*β-*FeSi_2_ structures with sufficient photodetection properties in the NIR can open up the possibility of designing opto-couples and lead to advanced silicon optoelectronics. Moreover, *β*-FeSi_2_ is compatible with the current Si technology, and both Fe and Si are abundant in the Earth’s crust^13^.

Unfortunately, most of the research teams emphasize that two main factors, associated with trap centres for photogenerated carriers, limit the performance of the Si/*β*-FeSi_2_ photodiode structure. The first of these factors is the diffusion of iron atoms into the silicon substrate during *β*-FeSi_2_ formation, which results in deep level formation^14^, and the second is the existence of dangling bonds in the heterojunction interface and initial *β*-FeSi_2_ layer, which causes the interface states^15^. In particular, the heterojunction interface between the *β*-FeSi_2_ film and the Si substrate has dangling bonds due to both grain boundaries and a lattice mismatch^16^.

In this work, we demonstrate an approach to spectral expansion of the silicon *p-n* junction photodetecting properties in the NIR by embedding *β*-FeSi_2_ nanocrystallites (NCs) into a *p*-type silicon layer. To reduce iron diffusion into the substrate, the deposition of thin 0.4 nm Fe layers is performed at room temperature. A multilayered structure obtained in this way would contain at most several nanometres of Fe, while for the same photodetection performance on *β*-FeSi_2_ films it is necessary to use hundreds of nanometres. In addition, heteroepitaxial stress may result in changes of the optical properties, band gap, and even electron dispersion law of the embedded *β*-FeSi_2_^17^. It was shown that photovoltaic properties of Si/*β*-FeSi_2_ film heterostructure are strongly limited by the diffusion length of the minority carriers in the *β*-FeSi_2_ absorbing layer, resulting in decreasing photoresponse with increasing film thickness^18^. To circumvent these problems, we put forward an idea to replace *β*-FeSi_2_ films with *β*-FeSi_2_ NCs, which, as we suggested, can be epitaxially embedded into the silicon matrix due to enhanced elasticity, which is typical for nano-size materials. In our previous work we also demonstrated that a light-emitting silicon diode with embedded *β*-FeSi_2_ NCs has a high-quality defect-free structure^19^,^20^, so photogenerated carriers from *β*-FeSi_2_ NCs move through the defect-free silicon layer. All of the abovementioned factors provide grounds for applying a silicon-silicide approach for the development of opto-couples.

This work follows the first study of photoelectrical properties of similar diode structures in open-circuit voltage mode^21^ that was performed earlier by our group and in photocurrent mode at low temperature^22^. We showed the possibility of expanding the spectral sensitivity into the NIR region.

## Results

**Figure 1:**
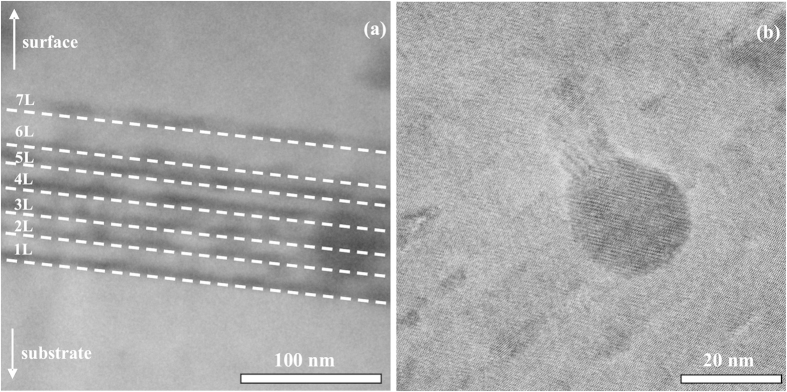
Cross-sectional TEM images of the seven-layered Si/*β*-FeSi_2_ NCs/Si structure. (**a**) A general view. (**b**) HRTEM image indicating the presence of two types of *β*-FeSi_2_ NCs. Dashed lines indicate the borders of the layers.

Figure 1 shows a cross-sectional Transmission Electron Microscopy (TEM) image of the grown sample. A well-defined multilayered structure with *β*-FeSi_2_ NCs was observed. The High Resolution Transmission Electron Microscopy (HRTEM) image denotes the existence of two types of NCs. The first type is the small NCs, several nanometres in diameter, with slightly elongated shape, which are located strictly at the depth of the Fe layer epitaxy. The first type dominates on the TEM images. The second type is spherical shape NCs, with a diameter of about 15–20 nm, occupying two or more layers. It is obvious that embedding of *β*-FeSi_2_ NCs has not caused the formation of any significant defects in the silicon layers, despite the lattice mismatch between the materials. As a consequence, there are no mismatch dislocations at the heterointerface. It can be assumed that the absence of the linear defects resulted from the small size of the NCs. In this case it is possible to accumulate elastic deformation in NCs rather than change to a relaxed state accompanied by the generation of defects. This situation is quite similar to the pseudomorphic growth of the thin films.

**Figure 2:**
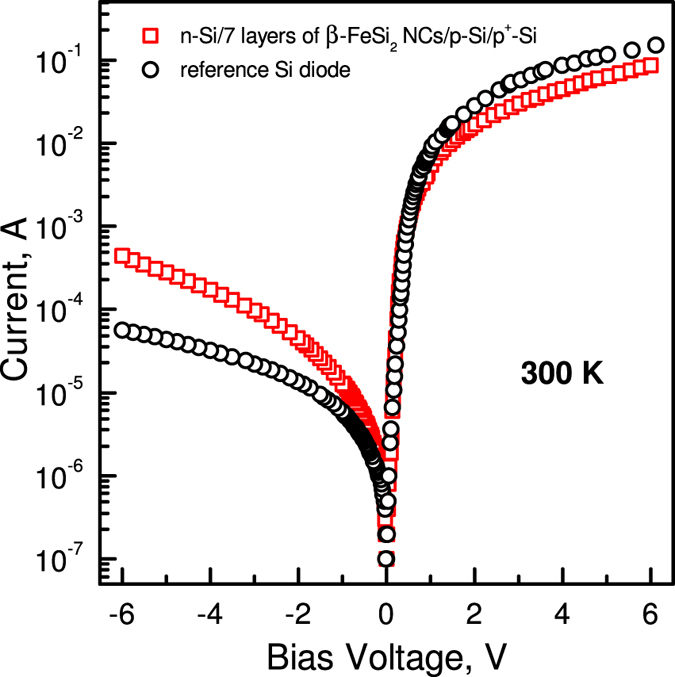
*I-V* characteristics of Si/*β*-FeSi_2_ NCs/Si and reference diode structures measured in the dark condition at room temperature.

Figure 2 shows the room temperature *I-V* characteristics of the Si/*β*-FeSi_2_ NCs/Si photodiode structure and reference Si *p-n* diode. The diodes show good rectifying behaviour, as expected, for the conventional Si ones: the forward current is more than approximately two orders of magnitude greater than that under reverse bias. Estimated values of the series and shunt resistances are 42 Ω and 100 kΩ respectively for the photodiode structure containing *β*-FeSi_2_ NCs, while in the case of the reference diode they are 30 Ω and 180 kΩ. Both diodes demonstrate nearly the same reverse saturation current of 0.11 μA and ideality factor of 1.

**Figure 3:**
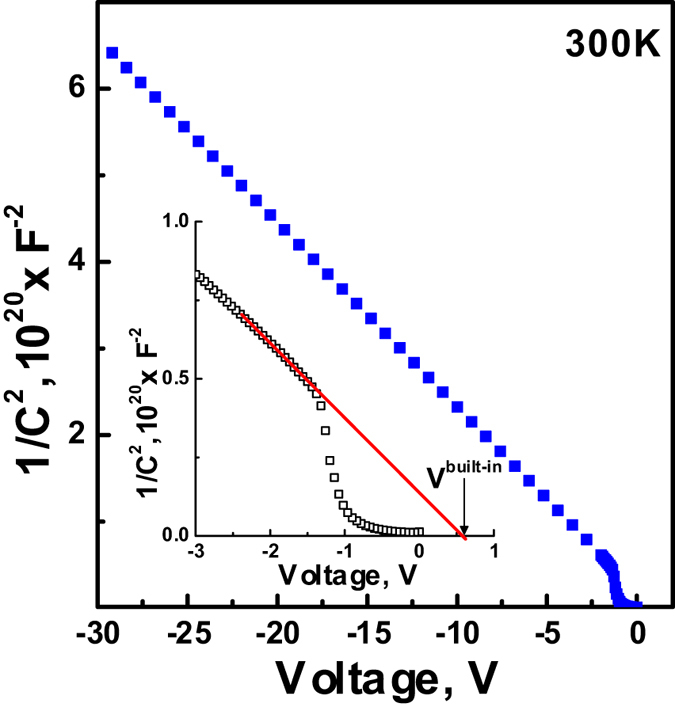
*1/C*^*2*^*-V* characteristic of Si/*β*-FeSi_2_ NCs/Si photodiode structure measured in the dark condition at 300 K. The intersection point of the red line with the voltage axis corresponds to the value of the built-in potential of the Si *p-n* junction at given doping levels and temperature.

The dependencies of *1/C*^*2*^ versus *V* (Fig. 3) showed that regardless of the presence of the NCs the value of built-in potential of the Si/*β*-FeSi_2_ NCs/Si structure along with the reference diode is 0.54 eV, which is in good agreement with the value for a conventional Si *p-n* junction at room temperature, at the given doping levels of the Si substrate (*N*_*d*_ = 5 × 10^14^ cm^−3^) and epitaxial *p*-Si layers (*N*_*a*_ = 1 × 10^14^ cm^−3^). Furthermore, on the *1/C*^*2*^ curve there is a linear part in the reverse bias range from –30 to –1 V, suggesting that it is due to the reduction of the interface state of the mesa diode^15^. From the foregoing, one can conclude that embedding of *β*-FeSi_2_ NCs into the depletion region of the Si *p-n* junction does not cause the formation of a significant amount of interface states.

At room temperature, the current under illumination is only slightly greater than the dark current, which can be associated with the relatively small amount of the *β*-FeSi_2_ (seven layers with only 0.4 nm of Fe in each) in comparison with the *β*-FeSi_2_ films on Si substrates, which show one order of magnitude difference between dark current and current under illumination^15^,^23^,^24^,^25^,^26^. On the other hand, embedded *β*-FeSi_2_ NCs form mainly scattered layers (Fig. 1) in silicon matrix, with a surface density of 10^11^ cm^−2^ at most, as was shown in our previous works^20^,^27^. Thus, the part of the NIR light passes through the structure without photocarrier generation.

**Figure 4:**
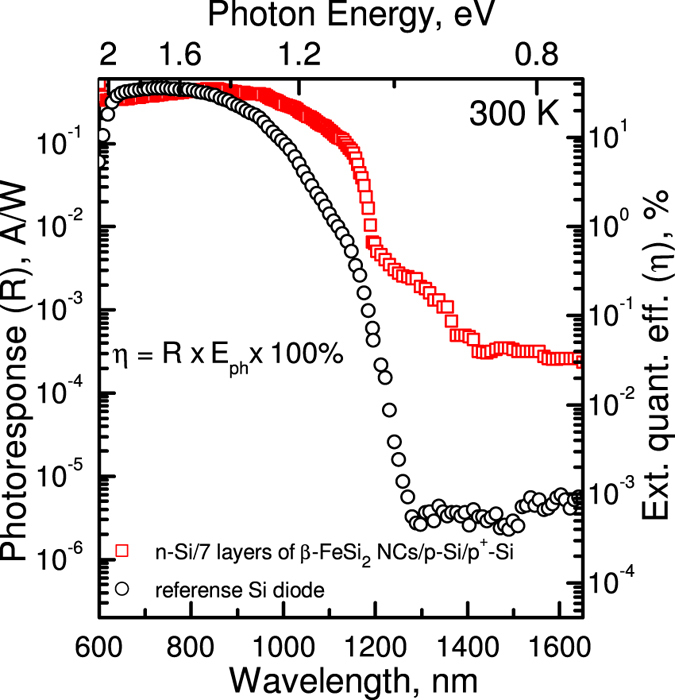
Photoresponse spectrum of the Si/*β*-FeSi_2_ NCs/Si diode measured at room temperature under zero bias conditions (circles) and calculated spectrum of the external quantum efficiency in comparison with the reference Si *p-n* junction (squares).

Measured photoresponse spectra of the structures at room temperature and zero bias are presented in Fig. 4. It is obvious that embedding of only seven layers of *β*-FeSi_2_ NCs into the silicon *p-n* junction resulted in: (i) enhancement of the spectral range of photosensitivity up to 1600 nm (0.77 eV); (ii) the photoresponse (*R*) being enhanced by more than two orders of magnitude at the telecommunication wavelengths; (iii) an almost ten-fold increase in photoresponse close to the silicon absorption edge (1000–1200 nm) as a result of the contribution of *β*-FeSi_2_ NCs.

## Discussion

The Selected-Area Fast Fourier Transformation (SAFFT) was applied to analyse the phase composition of the sample. The SAFFT for the type I NCs (see Methods Section) reveals, besides the typical Si patterns, four pairs of spots corresponding to *β*-FeSi_2_ planes with interplanar distances of 7.72 Å, 2.06 Å, 1.97 Å and 2.36 Å. These systems of planes are identified as (010) or (001) because of the large similarity between the b and c lattice constants of the *β*-FeSi_2_, (201), (331), and (312), respectively. The angle between *β*-FeSi_2_ (010)/(001) and Si (111) planes is 1.5°, suggesting almost coherent epitaxy with a corresponding epitaxy relationship or the formation of a low-angle boundary between the NCs and Si matrix.

The same measurements for the type II NCs represented in Fig. 1b (see the “Methods” section) confirmed that they correspond to *β*-FeSi_2_ phase as well. There are three identified interplanar distances of 4.91 Å, 4.80 Å and 3.89 Å that are coincident to the (100), (111), and (010) or (001) planes, respectively. The angle between the *β*-FeSi_2_ (100) and Si (110) planes is 3°. Thus, for the type II NCs, as well as for the small one, almost coherent epitaxy with the silicon matrix or low-angle boundary case takes place. The difference in the epitaxial relationships, in our opinion, is attributed to the difference in sizes of the NCs.

**Figure 5:**
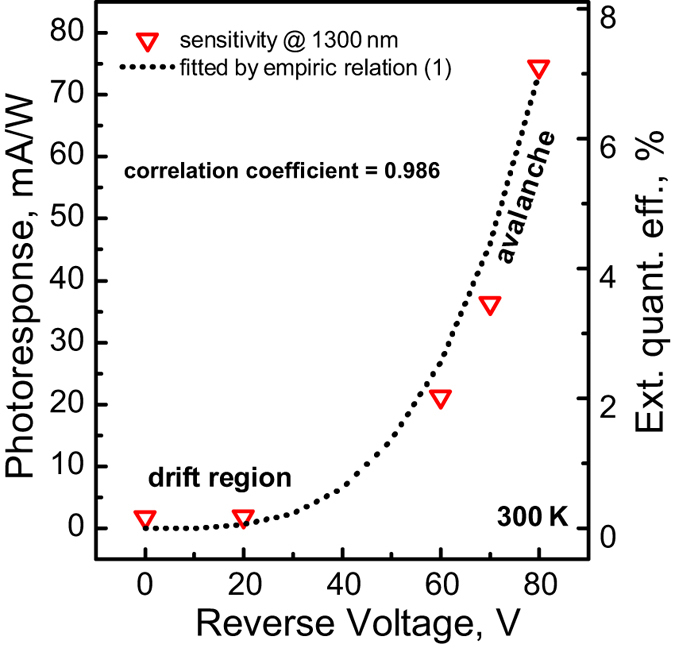
Experimental responsivity–reverse voltage dependence of the Si/*β*-FeSi_2_ NCs/Si photodiode fitted by the empirical relation.

Based on the measured photoresponse, we estimated the spectral dependence of the external quantum efficiency (*η*) of our device and reference diode following Sze^28^. The device at zero bias conditions demonstrates a room temperature external quantum efficiency of near to 0.2% at 1300 nm (Fig. 4). The relatively low photocurrent can be explained if one takes into consideration the small amount of the material capable of absorbing NIR light, which results in NCs contributing poorly to the generation of photocarriers. That is why we had to use a structure similar to *n-i-p* in order to multiply carriers applying reverse bias, as the absorption of NIR light in *β*-FeSi_2_ NCs would result in the generation of carriers which diffuse into the silicon, causing the development of an electron avalanche^29^. The existence of the avalanche process is also confirmed by an exponential growth of the photoresponse when the reverse bias exceeds 50–60 volts (Fig. 5), which corresponds to the avalanche mode. According to Fig. 5, the reverse bias dependence of the photoresponse at 1300 nm is fitted well by the empirical relation^30^ for the avalanche multiplication process:


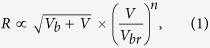


where *V*_*b*_ is the built-in voltage of the Si *p-n* junction, *V*_*br*_ is the breakdown voltage, and *n* is 3 for the *p*-type of the diode base^31^. The efficiency increases via avalanche multiplication in addition to the voltage root-square dependence of the *p-n* junction depletion layer. In our device we obtain a breakdown voltage of 54 V, a responsivity of 20 mA/W, and a value of the avalanche gain of 5. The maximum value of the external quantum efficiency obtained for the device is about 7%. From the analysis of the expression, it follows that: (i) the decrease of the breakdown voltage will result in a significant increase of the photoresponse, and (ii) it is possible to slightly increase the photoresponse by increasing the value of the built-in potential, which is restricted by the band gap of Si. The former can be achieved by changing the conduction type of the diode base to the *n-*type, which corresponds to *n* = 5 in the empirical expression^31^. In addition, a decrease in the thickness and dopant concentration of the Si layer (105 nm and *N*_*a*_ = 1 × 10^14^ cm^−3^ for the current device) reduces the breakdown voltage.

At room temperature, the photoresponse and external quantum efficiency values at the wavelength of 1300 nm in the avalanche mode (*R* = 20 mA/W; *η* = 2%) are quite comparable with those of *β*-FeSi_2_ thick films on Si (*R* = 3–15 mA/W; *η* = 1–2%)^23^,^24^,^25^,^26^ and even exceed the values obtained for a *β*-FeSi_2_ homojunction^32^. At the same time, we took advantage of the conventional Si *p-n* junction and expanded the functionality of the silicon diode structure in NIR photodetection, increasing the photoresponse by more than two orders of magnitude at important wavelengths for optical communications by adding a small amount of Fe to the initial system, while for the same results it is necessary to use films of *β*-FeSi_2_ (90–100 nm of Fe)^23^,^24^,^25^,^26^. Moreover, it was shown that embedding of *β*-FeSi_2_ NCs resulted in an increase of the sensitivity at the silicon absorption edge by more than one order of magnitude. It is interesting to note that the proposed approach gave rise to an increase in the integrated value of external quantum efficiency in the spectral range of 600–1700 nm from 18.7%, in the case of the reference Si photodiode, up to 23.3% for the *p*^+^-Si/*p*-Si/*β*-FeSi_2_ NCs/*n*-Si photodiode at zero bias. This fact suggests that embedding of *β*-FeSi_2_ NCs into Si may also result in increasing performance of the conventional silicon solar cells.

**Figure 6:**
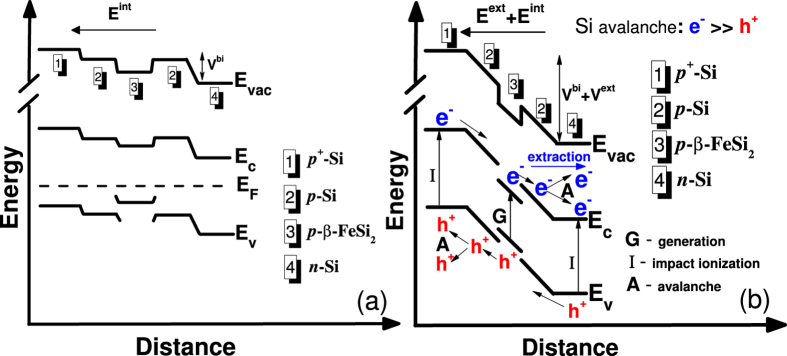
Band diagram of the Si/*β*-FeSi_2_ NCs/Si diode structure in equilibrium and nonequilibrium states.

To explain the gathering process of photogenerated carriers we propose a band diagrams of the diode structure in equilibrium and nonequilibrium states (Fig. 6). After the generation of carriers in the NCs, they diffuse into the *p*-type silicon layers, where the built-in potential of the Si *p-n* junction separates them. Then acceleration of the carriers by an external electric field takes place and the carriers may get sufficient energy for impact ionization of the silicon atoms, thereby developing an avalanche.

While constructing the band diagram in equilibrium state, we supposed that the applied bias drops entirely at the Si *p-n* junction rather than at the Si/*β*-FeSi_2_ heterojunction. This assumption is confirmed by calculation of the relation between relative built-in voltages in each of the semiconductors expressed by the formula^33^:


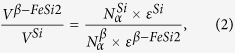


where *V*^*i*^ is the relative built-in voltage, *N*_*a*_^*i*^ is the concentration of the acceptors (10^14^ and 2–3 × 10^17^ cm^−3^ for Si and *β*-FeSi_2_, respectively), and *ε*^*i*^ is the dielectric constant of the materials (11.8 for Si^34^ and 27.6 for *β*-FeSi_2_^35^). We speculated that *β*-FeSi_2_ NCs formed by SPE – as well as thin *β*-FeSi_2_ films grown on Si in the same way – possess *p*-type conductivity with a carrier concentration of about (2–3) × 10^17^ cm^−3^ 36. Thus, the relation between relative built-in voltages is about 1 to 4000, suggesting that the applied bias drops at the Si *p-n* junction and does not cause a change in the energy level differences and barrier height at the *β*-FeSi_2_/Si heterojunction in equilibrium state. The values of the work function of Si and *β*-FeSi_2_ needed to construct the band diagram were taken from the literature data^34^,^37^, and the Fermi level positions depending on temperature and doping level were calculated following Sze^27^ using the value of the density of states in the valence band of *p-*type *β*-FeSi_2_ obtained by Kasaya *et al.*^38^.

Under the assumption of the low background photon flux at the zero bias voltage, following Keyes we calculated the Johnson-noise-limited specific detectivity (D*), which limits the noise performance of the infrared photodiodes^39^. The obtained device specific detectivity at 300 K in zero bias conditions is 1.2 × 10^9^ cm × Hz^1/2^/W at a wavelength of 1300 nm. The calculated value exceeds the results for *β*-FeSi_2_ films on average^15^,^23^,^24^ and is inferior in only in comparison with a passivated Si/*β*-FeSi_2_ heterostructure^25^.

## Conclusion

In summary, we fabricated a Si/*β*-FeSi_2_ NCs/Si photodiode structure grown by a combination of SPE for the formation of nanocrystallites with MBE for the epitaxy of the silicon capping layers. TEM investigations confirmed a well-defined multilayered structure with two types of embedded *β*-FeSi_2_ NCs. SAFFT verified the presence of semiconducting *β*-phase. Depending on the type of NCs, either *β*-FeSi_2_ (010)/(001) and Si (111) or *β*-FeSi_2_ (100) and Si (110) epitaxy is observed with a misalignment angle of 3° at most, corresponding to the almost coherent or low-angle boundary case. Despite the lattice mismatch between materials, there are no mismatch dislocations at the heterointerface even in the case of type II *β*-FeSi_2_ NCs.

*I-V* and *C-V* characteristics confirmed that embedding of the *β*-FeSi_2_ NCs into the depletion region of the silicon photodiode does not substantially influence the diode rectifying properties, the reverse saturation current, or the formation of the additional significant concentration of trap centres resulting from the interface states or diffused iron atoms. We suppose that it is related to non-defect epitaxy and embedding of the small-sized NCs into the silicon matrix together with implementation of the SPE, which restricted iron diffusion to the silicon substrate to some extent and contained the formation of deep levels associated with it.

On the strength of the obtained results, we suppose that the fact that the values of photoresponse and external quantum efficiency of our device are comparable with those for *β*-FeSi_2_ films is likely to be due to the defect-free heterointerfaces between the NCs and Si matrix, which results in a lower density of trap centres for photogenerated carriers and the advantages of avalanche multiplication.

Further improvements of our device may consist of the optimization of *p-n* junction parameters to operate at zero or small reverse biases. Another way to improve the performance, especially in the field of solar cells, is by embedding the NCs of different narrow band gap semiconducting silicides, for example CrSi_2_ (0.35–0.37 eV)^40^,^41^ of Ca_3_Si_4_ (0.35–0.63 eV)^42^, together with *β*-FeSi_2_ to cover both the NIR and the short wave infrared region (1.4–3 μm) of the solar spectrum.

## Methods

### Sample growth

The sample was grown on *n*-type (7–10 Ω × cm) monocrystalline silicon substrate with (111) orientation. *β*-FeSi_2_ NCs were formed by solid phase epitaxy (SPE) of 0.4 nm Fe at 630 °C followed by molecular beam epitaxy (MBE) of a thin (15 nm) weakly doped (*N*_*a*_ = 1 × 10^14^ cm^−3^) *p*-type Si layer at 750 °C. To obtain a multilayer structure, the SPE and MBE steps were repeated seven times, resulting in a total thickness of about 105 nm for the Si layer with embedded NCs (active region). The formation of the last covering layer was followed by the deposition of a *p*^+^-type Si (*N*_*a*_ = 10^17^ cm^−3^) layer (200 nm) at 750 °C to form an *n-i-p* structure, to some extent, and to achieve the ohmic contact with the Al contact layer. More detailed information about the growth procedure can be found in our previous works^20^,^21^. Next, the sample was chemically treated to form a mesa diode for photovoltaic measurements. Finally, Au-Sb alloy and Al were deposited to form contacts with *n*-Si and *p*-Si, respectively. For comparison, a similar diode structure was formed without NCs on the same substrate using the same deposition technique (referred to as the reference diode), with the only difference being in the thickness of the *p*-type epitaxial layer (600 nm).

### Crystal structure analysis

The study of the structure and morphology of nanocrystallites was performed using a JEOL-4000EX electron microscope operated at 400 kV, which is characterized by a spatial resolution by points to point of 0.16 nm and by lines of 0.1 nm, and a TITAN 80–300 instrument equipped with a spherical aberration corrector of the objective lens and an electron beam monochromator and characterized by a spatial resolution of 0.08 nm. The (110) cross-sections for HRTEM studies were prepared in a standard manner by etching with argon ions. The Digital Micrograph software (GATAN) was used for digital processing of experimental HRTEM images.

### Selected Area Fast Fourier Transforms

**Figure 7:**
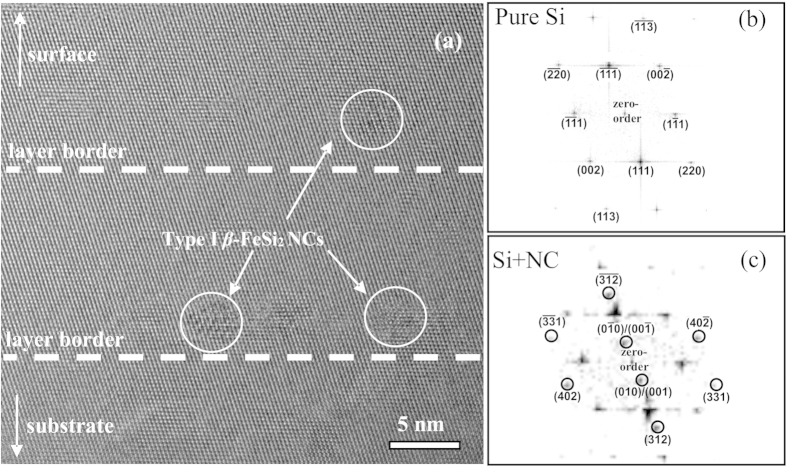
(**a**) HRTEM image of the Si/*β*-FeSi_2_ NCs/Si structure showing type I *β*-FeSi_2_ NCs, which are circled in white. Dashed lines indicate the borders of the layers. (**b**) SAFFT of the pure Si region. (**c**) SAFFT of the region containing *β*-FeSi_2_ NC. All the reflexes differing from that of Si are circled in black and identified.

**Figure 8:**
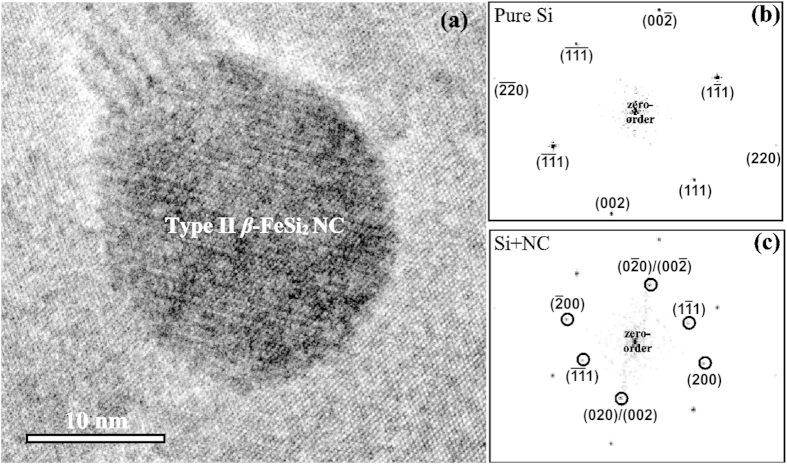
(**a**) HRTEM image of the Si/*β*-FeSi_2_ NCs/Si structure showing type II *β*-FeSi_2_ NC. (**b**) SAFFT of the pure Si region. (**c**) SAFFT for the region containing *β*-FeSi_2_ NC. All the reflexes differing from that of Si are circled in black and identified.

were performed using ImageJ software. The accuracy in determining the interplanar spacings was 0.05 nm, and calibration was carried out by well-known Si reflexes. Crystallographic planes were used as identifiers in all the SAFFT images; fold reflexes were not displayed in order to avoid cluttering the figures. Figures 7 and 8 show cross-sectional HRTEM images (a), SAFFT images of pure Si matrix (b), and SAFFT images of the type I and type II *β*-FeSi_2_ NCs (c), respectively. When identifying reflexes, in addition to comparing the estimated interplanar distances, the angles between crystallographic planes, which are material independent, were checked for accordance. Therefore, we managed to eliminate from consideration other silicide phases such as Fe_3_Si, ε-FeSi, α-FeSi_2_, or γ-FeSi_2_, which may have resulted in the presence of additional spots on the SAFFT images, which might be incorrectly taken as corresponding to *β*-FeSi_2_.

### Device characterization

Current-voltage (*I-V*) and capacitor-voltage (*C-V*) characteristics of the mesa diode were measured on an E7-20 immittance meter in the dark and under illumination conditions. Photoresponse measurements were carried out using a calibrated tungsten lump as a light source and a monochromator (Solar Tii, MS3504i) by the standard lock-in technique (Stanford Research Systems, DSP Lock-in amplifier SR830) with an optical chopper (f = 882 Hz). All the spectra were registered in a photocurrent mode.

## Additional Information

**How to cite this article**: Shevlyagin, A.V. *et al.* Enhancement of the Si *p-n* diode NIR photoresponse by embedding *β*-FeSi_2_ nanocrystallites. *Sci. Rep.* **5**, 14795; doi: 10.1038/srep14795 (2015).
